# A systematic review: effectiveness of mass media campaigns for reducing alcohol-impaired driving and alcohol-related crashes

**DOI:** 10.1186/s12889-015-2088-4

**Published:** 2015-09-04

**Authors:** Rajendra-Prasad Yadav, Miwako Kobayashi

**Affiliations:** Stop TB Unit, World Health Organization Representative Office in Cambodia, No 177-179 Street Pasteur and 254, Sangkat Chak Tomouk, Khan Daun Penh, Phnom Penh Cambodia

## Abstract

**Background:**

Mass media campaigns have long been used as a tool for promoting public health. In the past decade, the growth of social media has allowed more diverse options for mass media campaigns. This systematic review was conducted to assess newer evidence from quantitative studies on the effectiveness of mass media campaigns for reducing alcohol-impaired driving (AID) and alcohol-related crashes, particularly after the paper that Elder et al. published in 2004.

**Methods:**

This review focused on English language studies that evaluated the effect of mass media campaigns for reducing AID and alcohol-related crashes, with or without enforcement efforts. A systematic search was conducted for studies published between January 1, 2002 and December 31, 2013. Studies from the review by Elder et al. were added as well.

**Results:**

A total of 19 studies met the inclusion criteria for the systematic review, including three studies from the review by Elder et al. Nine of them had concomitant enforcement measures and did not evaluate the impact of media campaigns independently. Studies that evaluated the impact of mass media independently showed reduction more consistently (median −15.1 %, range −28.8 to 0 %), whereas results of studies that had concomitant enforcement activities were more variable (median −8.6 %, range −36.4 to +14.6 %). Summary effects calculated from seven studies showed no evidence of media campaigns reducing the risk of alcohol-related injuries or fatalities (RR 1.00, 95 % CI = 0.94 to 1.06).

**Conclusions:**

Despite additional decade of evidence, reviewed studies were heterogeneous in their approaches; therefore, we could not conclude that media campaigns reduced the risk of alcohol-related injuries or crashes. More studies are needed, including studies evaluating newly emerging media and cost-effectiveness of media campaigns.

## Background

The World Health Organization (WHO) estimates that the number of people killed in road traffic crashes is about 1.2 million per year, and the number injured is as high as 50 million per year [1]. Over 90 % of road traffic deaths occur in low-income and middle-income countries. Alcohol is found to be present in 33–69 % of fatally-injured drivers, and 8–29 % of non-fatally injured drivers [2].

Mass media campaigns has long been used as a tool for promoting public health, and their effectiveness have been assessed and described in different literature [3]. Some studies linked with successful campaigns are those focusing on adoption of new behaviors as compared with prevention or cessation of problem behaviors, or those that had concomitant law enforcement aspects [3, 4]. Among media campaigns focusing on prevention or reduction of substance use, data shows that campaigns focusing on alcohol use may be more successful than campaigns focusing on illicit drugs or tobacco [3, 5].

Many countries around the world have been using the triangle of legislation-enforcement-publicity for effective social marketing campaigns against alcohol-impaired driving (AID) [6]. High visibility enforcements of legislation generally utilize a combination of high-fear emotive advertising to change attitude and low-fear informational advertising to change knowledge [7]. In 2004, Elder et al. published a systematic review on the effectiveness of mass media campaigns for reducing AID and alcohol-related crashes [8]. The results showed that, overall, media campaigns lead to a median decrease in alcohol-related crashes of 13 % (interquartile range: 6 to 14 %).

Traditionally, media have been categorized into three types: paid, earned, and owned [9]. *Paid media* include traditional advertising, where an advertiser pays for space or for a third party to promote something that the advertiser wants to draw attention to. Examples include TV commercials and magazine and newspaper advertisements. *Earned media* are publicity you get for free such as by news coverage or when the public spread information through external or their own media at no cost to yourself. *Owned media* consists of properties or channels owned by the advertiser that uses them for the purpose of promotion. Examples include websites or brochures created and owned by the advertiser. Mass media campaigns have usually used a combination of these media types. During the past decade, the Internet has rapidly developed, and social media have become one of the most popular Internet services in the world [10]. It has been used in health promotion campaigns as well, although reports have shown variable outcomes [11–13]. With the availability of wider options to deliver media campaigns, we considered that new evidence might be available in the effectiveness of mass media campaigns in reducing AID since the paper that Elder et al. published in 2004.

## Objectives

**Fig. 1:**
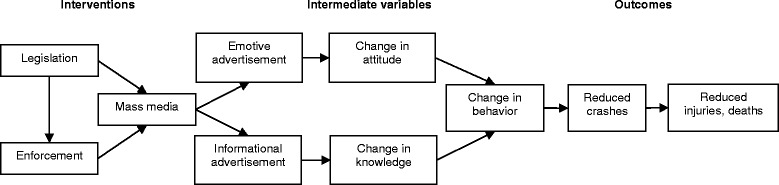
Logical framework of causal relationships between different types of interventions and road crashes

The primary objective of this systematic review is to assess available evidence from quantitative studies after the review by Elder et al. [8] on the effectiveness of mass media campaigns with or without concomitant enforcement activities for reducing AID and alcohol-related crashes compared to no media interventions among drivers of any type of motor vehicle on public roads in any country, state, or community. See the logical framework in Fig. 1, which guided the review.

## Methods

### Eligibility criteria

*Types of studies* included experimental, quasi-experimental and observational. The language was limited to English. Only papers published after January 2002 were considered. Population included all drivers of any type of motor vehicle on public roads, of any gender and of all ages. Any lengths of follow-up were included. *Settings* included any country, state or community of any size. *Interventions* included any type of mass media used for reducing AID, with or without enforcement efforts. *Comparators* included any type of control or comparison group or area not exposed to the campaign and with no changes in legislation, enforcement or publicity during the period of the study. Studies without comparator groups were also included.

*Primary outcome measures* included alcohol-related crashes and alcohol-related crash injuries and fatalities. *Secondary outcome measures* were used as surrogates for primary outcome measures but only if the latter were unavailable. These included single-vehicle-night-time crashes, all nighttime crashes, all single vehicle crashes and all crashes. Blood alcohol concentrations measured at sobriety points and interview reports of target populations were excluded as outcome measures because of the potential to be biased due to police’s and target populations' knowledge of the intervention, respectively [14]. If the study did not provide specific figures for the outcome measures, it was excluded from analysis.

### Search strategy

The review searched the following computerized databases: PubMed, Ovid Medline, EMBASE, Psych Info, Transport Research International Documentation (TRID), Scopus, and Global Health.

The search syntax that this review used was: (mass media or television or TV or radio or cinema or movie* or film* or social media or social network* or publicity campaign or campaign* or market*) and ((alcohol or beer or wine or spirit*) and (drink* or intoxicat* or intake or consum*) and (automobile* or car or cars or road or traffic or truck* or driving or driver*) and (crash* or accident* or collision*). In addition to this syntax, the review ‘exploded’ database-specific MeSH terms if the databases supported this.

The searches were limited to publications in English language. Since this review was intended to be an update of the review by Elder et al. in 2004 [8], which had reviewed relevant studies published until 31 December 2001, the literature searches for this review were set from January 1, 2002 to “current” (31 December 2013). In addition, this review included all studies of Elder 2004 [8] except the study by McLean et al. [15] which had an outcome measure of blood alcohol concentration, which does not fit the eligibility criteria of this review.

### Study selection

The two reviewers (MK and RY) independently examined titles, abstracts and key works of citations from electronic databases for eligibility. The reviewers tried to err on the side of over-inclusion during this stage. For studies that appeared to meet the inclusion criteria, or in cases when a definite decision could not be made based on the title or abstract alone, the full text were obtained for detailed assessment against the inclusion criteria. For manuscripts that could not be obtained, an attempt was made to contact the authors for information. Studies were excluded at this stage if they failed on one or more criteria. Reasons were recorded for the exclusions. The selection was done using the software EPPI-Reviewer 4, version 4.3.6.0.

### Data collection process and data items

Once studies were selected, data was extracted using a standard form developed for this review. Extracted data items included study objectives, methods, participants, follow-up period, settings, interventions, and outcomes.

### Summarizing outcome measures

Whenever available, alcohol-related fatal crashes were used as the outcome and figures between the intervention group and control group were compared. Unless a model was used (e.g. regression models or Autoregressive Moving Average Model (ARIMA) for interrupted time series (ITS) studies) that calculated the degree of change during the study period, the changes in individual studies were calculated using the difference in pre- and post- intervention means. The summary effects from all included studies were described using the median and the range. Since proxy measures were used in some studies, the following order of priority was used to select the outcome measures: single-vehicle-night-time crashes, all-night-time crashes, all-single vehicle crashes, and all crashes. Use of fatal crashes were given priority over nonfatal injury crashes, as fatal nighttime crashes is considered as a validated surrogate for alcohol-related fatalities [16].

### Summary effects measures calculation

Given the heterogeneity in the outcome measures used in the studies, for the purpose of summary effects measures calculation, we selected studies that allowed us to calculate the relative risk of alcohol-related fatal crashes among all crashes pre- and post-intervention. If information on total number of crashes was not available, alternative measurements (e.g. fatal crashes among drivers in the campaign target population with BAC ≥0.08 g/dL versus all alcohol-related fatalities) were selected to help control for the overall trend in total crashes and other factors that may influence the total number of crashes [8]. We estimated pooled relative risks using the random-effects model. Review Manager 5.2 (version 5.2.4) was used for this analysis.

### Assessment of risk bias

Assessment of the risk bias in individual studies at the study as well as outcome levels was done to determine the methodological quality of the included studies. For this purpose, this review used the guidelines provided by the EPOC checklist [17], and classified the studies into “good quality”, “intermediate quality” and “low quality”. If the study did not use any model for analysis, it was considered as a low-quality study. Also, if the measured effects were inclusive of interventions other than mass-media (e.g. other enforcement measures or educational activities), the study was considered as intermediate quality at its best.

## Results

### Study selection

**Fig. 2:**
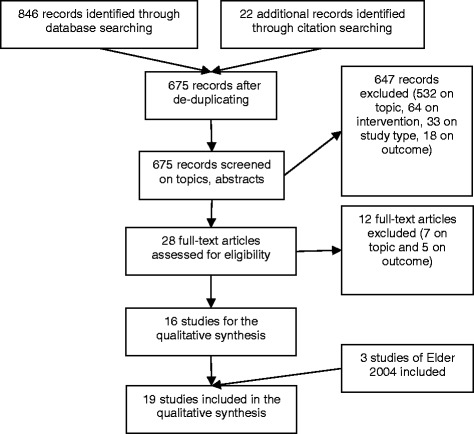
Flow diagram of study selection

See Fig. 2. All searches yielded 868 titles. First, all duplicates were removed to yield 675 studies. Thereafter, 647 studies were removed based on titles and abstracts to yield 28 studies. Full-text articles were reviewed for the 28 titles for topic, language, interventions and outcomes. This yielded 16 studies, and were included in this review in addition to three studies from Elder [8].

### Study characteristics

The study characteristics are summarized in Tables 1 and 2.

**Table 1:** Studies included based on eligibility criteria: studies with no increases in enforcement activities or with statistical models to account for those increases

Author, Year (study period) Objective, Design, Evaluation setting	Intervention Details: Scope (national, state, community) Message theme (tagline) Delivery method Cost Other details	Results/Other Information	Summary value	Follow-up period
Whittam 2006 [18]	Objective: Assess the potential impact of public-service assessments on young drivers between the ages of 16 and 19 years	All crashes among 16–19 year olds	Net change in serious-injury crashes among 16–19 year olds: 18.3 % decrease	4.5 months
CITS, 1994–1999				
Intervention period: Aug 15–Dec 31, 1996 (excluding Oct)				
		Intervention site:		
Intervention sites: Kingsport, Johnson City, and Bristol, Tennessee		• 21.6 % reduction during the intervention period (*p* < 0.05)		
Comparator sites: Hamilton County				
	Mass media: Paid television and radio announcements, billboard display	Comparator site:		
		• 3.2 % increase in crashes (*p* = 0.61)		
		Net difference:		
		• 24.8 %		
		Serious-injury crashes among 16–19 year olds		
		Intervention site:		
		• 16.4 % decrease (*p* = 0.19)		
		Comparator site:		
		• 1.9 % increase (*p* = 0.17)		
		Net difference: 18.3 %		
Murry 1993 [19]	Objective: Evaluate an anti-drinking and driving advertising campaign targeting 15–24 year olds.	Nighttime fatal and incapacitating accidents for 15–24 yo males and females (intervention site: −7.14 %, comparator: +11.8 %, *p* = 0.06)	Net change −18.9 % (*p* = 0.05)	6 months
Jan 1983–Sept 1987, monthly CITS				
Intervention: Wichita, Kansas, USA				
Comparator: Omaha, Nebraska, USA	Mass media: 6-month paid media schedule using television, radio, newspapers, and billboards			
Newstead 1995 [20]	Objective: Evaluate various safety measures implemented starting Sept 1989 in Victoria, Australia.	Contribution of drink-driving publicity in reducing nighttime serious casualty crashes: approximately 14 % (average of 1990–1992)	Reduction of nighttime serious casualty crashes in Victoria from 1990 to 1992 was 14 %	3 years
1983–1992				
ITS				
Intervention: Victoria, Australia				
Comparator: None				
	Mass Media:			
	TV advertising, Dec 1989 to Dec 1992, radio, press, outdoor advertising, Sky Channel and cinema	Statistically significant in Victoria (*p* < 0.05), but not in Melbourne crashes (*p* = 0.07)		
	Enforcement: Random breath testing, lowering of freeway speed limit, speed cameras			
Tay 2002 [21]	Objective: Evaluate New Zealand’s Supplementary Road Safety Package initiated by Land Transport Safety Authority in 1995	Estimated impact of the advertising campaign on the number of fatal crashes using regression model:	Estimated impact of advertising campaign on the number of fatal crashing has no impact on the target population (male 15–34 years old)	2 years
ITS, 1988–1996 (108 observations)				
Intervention site: New Zealand				
Comparator site: None				
		• Male drivers between 35 and 54: 29.91 % decrease		
		• Female drivers between 15 and 24: 40.21 %		
		• Female drivers between 25 and 34: 70.04 %		
	Media campaign: TV, mainly targeting			
		• No impact on young male drivers (15–34)		
	18–24 year olds			
	Enforcement:	Estimated impact of the program before and after implementation of the campaign:		
	Speed cameras, advanced speed detectors, compulsory breath testing			
		• Male drivers: −32.9 % (15–24yo) to +4.7 % (55 years and older)		
		Female drivers: −56.8 % (25–34 %) to −26.7 % (55 years and older)		
Jones 2005 [22]	Objective: Evaluate “Smart Roads” program in Pueblo, Colorado aimed at drivers aged 21–34.	Nighttime injury crashes decreased by 39 % in the intervention counties, whereas it increased by 3.3 % in the control counties (*p* < 0.0001)	Nighttime single-vehicle crashes: net change 28.8 %	4 years
Before: 1998 to 1999				
After: 2000 to 2001				
CBA				
Intervention group: Pueblo county (intervention site) plus eight other low-population surrounding counties	Mass media:			
	Television, radio, and newspaper advertisements, billboards, bumper stickers, bus station banners, other collaterals)	Nighttime single-vehicle crashes decreased by 24.8 % in the intervention counties, whereas there was a 4.0 % increase in the control counties (*p* = 0.01)		
Comparison: all other counties in Colorado	Workplace initiative education program.			
Epperlein 1987 [29]	Objective: Evaluate the effect of crackdown on drinking drivers in Arizona	Impact estimates of the anti-drunk-driving publicity campaigns of March, 1982	Nighttime fatal crashes (net change): −16.2 %	22 months
March 1972-Dec 1983 ITS				
Intervention site: Arizona, USA	Mass media:	• Nighttime fatal crashes −26.8 % (pre-intervention mean/month. 724)		
	Television, print, and radio advertisements, billboards, posters, bumper stickers (March 1982)			
Comparator site: None (daytime crashes and crashes with no identified drinking drivers used for comparison)				
		• Daytime fatal crashes −10.6 % (pre-intervention mean/month. 1633)		
	Enforcement: Stricter DWI legislation Increasing the minimum drinking age (August 1982)			
		Net change: −16.2 %		
		• Drinking drivers in crashes −14.0 % (pre-intervention mean/month. 1036)		
		• Non-drinking drivers in crashes −0.8 % (pre-intervention mean/month. 11345)		
		Net change: 13.2 %		
Zampetti 2013 [34]	Objective: To verify the effect of intensive vs. basic road safety education programs on the incidence and severity of nonfatal road injuries (NFRTI)	The number of NFRTI	Difference in incidence of NFRTI in the basic site: −0.04 % (*p* = 0.05)	5 years
Before: Jun–Aug 2003		• Before: 907,		
After: Jun–Aug 2008		After: 755		
CBA		Incidence of injuries in the basic campaigns (8 municipalities)		
Intervention period: 2003–2008				
Intervention sites: 20 municipalities in the Local Health Authority 1 (LHA1) area in Campania, Italy	Publicity campaigns: Billposting on public transport, bus stops, train stations, in bars and meeting places. Dispatch of brochures, pamphlets, and posters	• Difference in incidence of injuries −0.4 per 1,000 (2003 (before) 1.1, 2008 (after) 0.7)		
No comparator site				
	Mass media: press conferences, articles in local papers, radio/television interviews, and the LHA1 web site	• Incidence of injuries in the intensive campaigns (12 municipalities)		
	Sites for intensified approach (12 out of 20 municipalities):	• Difference −0.5 per 1,000; *p* < 0.001		
	School campaigns and community conferences, 1-day conference at the end of school year			
Worden 1975 (Elder) [35]	Objective: Evaluate Vermont public education campaign on alcohol and highway safety	The proportion of “high-risk” male drivers (those who report consuming three or more drinks at least once a week) above 0.05 g/dL BAC:	Drivers above 0.05 g/dL BAC: −158 %	24 months
May 1972–May 1974				
CBA				
Intervention site: Vermont	Mass media: Radio, TV, drive-in theater spots.		Fatal crashes: 0 %	
Comparison site: counties with no intervention		• At mid-campaign (May, 1973) decreased 37 % from a baseline of 10 of 48 drivers to 9 of 69 (95 % CI: −72 % ~ +42 %; net change = −158 %)		
	Enforcement: Stayed high throughout the study period			
		• Immediately following the campaign (May, 1974) decreased 67 % (95 % CI: −88 % ~ −7 %; net change −111 %)		
		The proportion of had-been-drinking to total fatal crashes decreased 6 % from a baseline of 9 of 20 to 8 of 19 (95 % CI: −54 % ~ +91 %; net change 0 %)		
		*Very small sample sizes		
Cameron 1998 (Elder) [36]	Objective: Evaluation of the first two years of the New Zealand Supplementary Road Safety Package that was introduced in 1995/1996 (supplements CBT and speed camera programs introduced in 1993)	In 1996–1997, campaign estimated to result in:	Injury crashes	24 months
Jan 1990–June 1997, quarterly			Arm 1 (Urban): −7 %	
CITS				
Intervention: New Zealand (crashes during high alcohol consumption hours)		• A 33 % decrease in urban high alcohol hour serious injury crashes (95 % CI: −40 % ~ −25 %; net change = −7 %)	Arm 2 (Rural): −18 %	
Comparator: New Zealand (crashes during low alcohol consumption hours)				
	Mass media: primarily TV advertising campaigns	• A 32 % decrease in rural high alcohol hour serious injury crashes (95 % CI: −41 % ~ −22 %; net change = −18 %)		
	Enforcement: Sobriety checkpoint			
		In 1995–1996, campaign estimated to result in:		
		• A 16 % decrease in urban high alcohol hour serious injury crashes (95 % CI: −24 ~ −6 %; net change = −2 %)		
		A 6 % decrease in rural high alcohol hour serious injury crashes (95 % CI: −18 % ~ −7 %; net change = −5 %)		

*BAC* Blood Alcohol Concentration, *CBA* Controlled Before-After, *CBT* Compulsory Breath Testing, *CI* Confidence Interval, *CITS* Controlled Interrupted Time Series, *DWI* Driving While Intoxicated, *ITS* Interrupted Time Series, *LHA* Local Health Authority, *NFRTI* Nonfatal Road Injuries, *NHTSA* National Highway Traffic Safety Administration, *TV*, Television, *USA* United States of America

**Table 2:** Studies included based on eligibility criteria: studies with increases in enforcement activities but without statistical models to account for those increases

Author, Year (study period) Objective, Design, Evaluation setting	Intervention details: scope (national, state, community) message theme (tagline) delivery method cost other details	Results/other information	Summary value	Follow-up period
Fell 2008 [23]	Objective: Evaluate the impaired-driving demonstration projects conduced in 7 states.	Indicators relative to surrounding states	Compared to surrounding states, Georgia, Tennessee, Indiana, Michigan had statistically significant decreases in the Ratio, whereas in some States (Louisiana, Texas), there were increases in the Ratio.	12–18 months
CITS		Ratio: ratio of drinking drivers (BAC > =0.01) to nondrinking drivers (BAC = 0.00) in fatal crashes		
2000–2003				
7 selected states in the US (Georgia, Louisiana, Pennsylvania, Tennessee, Texas, Indiana, and Michigan)	Use of paid media (+/− earned media):			
	Georgia, Louisiana, Tennessee, Texas, Indiana, Michigan	VMT: alcohol-related fatalities (driver or pedestrian total BAC > 0.01) per 100 million		
	Sobriety checkpoints:			
		VMT		
Comparator (within-state comparison: Georgia, Tennessee, Michigan; neighboring states: selected nearby states, pooled; the rest of the nation, pooled).	Georgia, Louisiana, Pennsylvania, Tennessee, Indiana	Georgia:		
	Saturation patrols:			
	Louisiana, Tennessee, Indiana, Michigan			
	Community education/partnership:			
	Pennsylvania, Michigan			
		Ratio: −14 % (*p* < 0.05), VMT −5 %		
		Louisiana:		
		Ratio: 1 %, VMT15% (*p* < 0.05)		
		Pennsylvania:		
		Ratio: −9 %, VMT: −2 %		
		Tennessee:		
		Ratio: −11 %(*p* < 0.035), VMT: 1 %		
		Indiana:		
		Ratio: −13 %(*p* < 0.018), VMT: −20 % (*p* < 0.002)		
		Michigan:		
		Ratio: −14 % (*p* < 0.07), VMT −18 % (*p* < 0.003)		
		Texas:		
		Ratio: 3 %, VMT: 5 %		
Zwicker 2007a [24]	Objective: Evaluate the effect of the National Highway Traffic Safety Administration impaired driving high-visibility enforcement model in 2002 in West Virginia	Alcohol-related fatalities in targeted counties: reduction of 0.99 lives each month. - 24 % (*p* = 0.01)	Alcohol-related fatalities in targeted counties: − 24 % (*p* = 0.012)	18 months
CITS				
2000–2004, monthly				
		Alcohol-related fatalities in targeted countries for men 21–34yo: reduction of 0.09 lives per month (*p* = 0.79)		
Intervention period: July 2003- Dec 2004				
Comparison period: Jan 2000- June 2003	Mass Media:			
	Paid media (TV)			
Intervention site: 6 counties in West Virginia	Enforcement:	Statewide alcohol-related fatality trend: reduction of 1.6 fatalities per month (*p* = 0.20)		
	Sobriety checkpoints, saturation patrols			
Comparator site: 49 non-targeted counties				
Zwicker 2007b [25]	Objective: Evaluate Connecticut’s statewide impaired-driving publicity and enforcement campaign	The overall alcohol-related fatality trend for the State:	Net change in alcohol-related fatalities in the state: −36.4 %	18 months
CITS				
Jan 2000- Dec 2004, monthly		Estimated reduction of 2.604 lives each month (*p* = 0.01) for the 18 mo. following the beginning of the campaign (Net change: lives saved during 18 mo., 36.4 % decrease)		
			Net change in alcohol-related fatalities among men 21–34 years old: −29.7 %	
Intervention phase: July 2003- Dec 2004, Comparison phase: Jan 2000- June 2003	Mass media:			
	Paid and earned media targeting men 18–34 years old			
	Enforcement:			
Intervention site: Connecticut, USA	Sobriety checkpoint	The alcohol-related fatality trend for fatalities involving men 21 to 34 years old:		
		Estimated reduction in the number of fatalities by 1.568 lives each month for the 18 mo. following the beginning of the campaign (*p* < 0.03) compared to 0.16 lives per month saved in contiguous counties (Net change: 25 lives saved during 18months, 29.7 %)		
Comparator site: 3 neighboring states				
Lacey 2008 [26]	Objective: Evaluate NHTSA Checkpoint Strikeforce program done July-December of each year, 2002–2004.	Alcohol-related fatal crashes in the intervention sites: −7.1 % relative to the nation as a whole (*p* = 0.119). In one State, West Virginia, the reduction was 16.7 % (*p* = 0.02) when compared to the Nation as a whole.	Alcohol-related fatal crashes: −7.1 %	3 years
CITS				
1991–2004, annually				
Intervention sites: Delaware, Maryland, Pennsylvania, Virginia, West Virginia, District of Columbia	Mass Media:			
	Paid and earned media. “Checkpoint Strikeforce. You Drink & Drive. You Lose.”			
Comparator: entire nation	Enforcement:			
	Checkpoints. BAC measurements (Maryland, Delaware, and Virginia)			
Agent 2002 [27]	Objective: Document	Number in 2002 compared to the average of the previous three years	Alcohol/drug related crashes: −9 %	4 years (13 days per year)
ITS	the results of the “You Drink& Drive.			
Before intervention: 13 days around Labor day in1999–2001	You Lose” campaign.	1. The number of crashes in which alcohol and/or drugs were listed as a contributing factor or the driver was noted to be suspected of drinking: −9 % (not statistically significant)	Number of injuries and fatalities resulting from alcohol/drug related crashes: −5 %	
	Enforcement:			
	Checkpoints and saturated enforcement activity			
Intervention: 13 days around Labor day in 2002	Mass media:			
	Paid media: broadcast and cable television, radio (from 15 to 30 Aug, 2002), and outdoor billboards (15 Aug–15 Sep, 2002)			
Intervention site: Kentucky, USA				
		2. The number of injuries and fatalities resulting from these crashes: −5 %		
Comparator: none				
Solomon 2008 [28]	Objective: Evaluate the effect of the National 2006 Labor Day holiday campaign, “*Drunk Driving. Over the Limit. Under Arrest*.” Targeting age group 18 to 34 years old	1. The total number of alcohol-related fatalities: 17,602 in 2006 compared to 17,590 in 2005 (0.07 %).	The total number of alcohol-related fatalities: 0.07 % increase (2005–2006)	4 months (Sep-Dec 2006)
ITS				
Intervention period: 3 weekends leading up to and around the Labor Day holiday period in 2006				
		2. The number of motor vehicle fatalities for male drivers (BAC 0.01 or higher) age 18 to 34: decreased from 5782 to 5654 (−2.21 %)		
	Mass Media:			
	1. Earned media (Aug 7- Sep 10)			
Intervention: 2006	2. Paid media (Aug 16–20; 23–27; Aug 30- Sep 3)			
		3. The number of motor vehicle fatalities for male drivers (BAC 0.08 or higher) age 18 to 34: decreased from 4996 to 4872 (−2.48 %)		
Comparaison: 2005				
	Enforcement:			
Intervention site: USA (nationwide)	Sobriety checkpoints, saturation patrols			
Comparator site: none				
Beck 2009 [31]	Objective: Evaluate	Net change in three-year averages before and during campaign in Maryland:	Alcohol-related total	6 years
			crashes: 2.2 %	
CITS	the effect of the Checkpoint Strikeforce campaign		Total alcohol-related fatalities: 14.7 %	
Before intervention: 1999–2001, Intervention: 2002–2004	Mass Media:	● Alcohol-related total crashes: 2.2 %	Alcohol fatalities as a percentage of total fatalities: Net change 3 %	
	Paid and earned media	● Alcohol-related injury crashes: −4.7 %		
	Enforcement:	● Alcohol-related fatality crashes: −2.7 %		
Intervention site: Maryland (Pennsylvania, Delaware, West Virginia, Virginia, District of Columbia)	Sobriety checkpoints			
		● Total alcohol-related fatalities: 14.7 %		
		● Alcohol-related injured drivers: −3.8 %		
Comparator sites: Minnesota, Oregon, and Washington				
Miller 2004 [30]	Objective: Evaluation of three incremental CBT program approaches	Mass media is estimated to have decreased in nighttime fatal or serious crashes decreased by 13.9 % (90 % CI = −26.1 to −0.1) nationally	Nighttime fatal or serious crashes: −13.9 %	10 years
ITS				
Intervention (CBT): 1993~				
Intervention (media): 1995~	Mass Media: National anti-drunk-driving campaign with hard-hitting messages			
Intervention (CBT enhancement): 1996~				
Intervention sites: New Zealand (CBT enhancement in Northern Police Region)	Enforcement: CBT checkpoints, (Northern Region) highly visible CBT through booze busses			
NHTSA 2007 [32]	Objective: Evaluation of the effect on the	Total declines in yearly average of fatal crashes for alcohol-impaired drivers from 2002 to 2005 were slightly greater for the non-SES, as compared with the SES (a 5 % drop in non-SES compared to a 2 % decline in SES, net decline: 3 %).	Net decline in yearly average of fatal crashes for alcohol-impaired drivers: −3 %	5 years
CITS	National Impaired Driving Crackdown Campaign targeting men 21 to 34 years old			
Before: 2001 and 2002				
After: 2004 and 2005				
Intervention sites: 13 Strategic Evaluation States (SES) (Alaska, Arizona, California, Florida, Georgia, Louisiana, Mississippi, Montana, New Mexico, Ohio, Pennsylvania, Texas, West Virginia)	Mass Media: paid and earned media (done nationwide). Additional advertising done in			
		In the target group of 18–34 year-old-male drivers, the decline was greater in non-SES compared to SES (8.7 % in non-SES and 3.8 % in SES).		
	SES.			
Comparator sites: non-SES	Enforcement: Sobriety checkpoints or saturation patrols in SES			
Suriyawongpaisal 2002 [33]	Objective: Evaluate the campaign against drink-driving and enforcement efforts	Percentage of the traffic injury victims who were drivers with illegal BAC (0.05 or more): 14.6 % increase in 9 months (*p* = 0.20)	Percentage of the traffic injury victims who were drivers with illegal BAC (0.05 or more): 14.6 % increase	9 months (assessed in alternating months)
ITS				
March-Nov 2002, alternating months	Mass Media:			
Intervention sites: 4 of the 21 public hospitals in Bangkok, Thailand				
Comparator site: None				
	Active public education program at national scale (roadside posters; bumper; radio and TV programs or spots; public announcements; press reports), 1997			
	Enforcements:			
	Highly visible sobriety check points, 1999			

*BAC* Blood Alcohol Concentration, *CBT* compulsory breath testing, *CITS* Controlled Interrupted Time Series, *ITS* Interrupted Time Series, *NHTSA* National Highway Traffic Safety Administration, *SES* Strategic Evaluation States, *VMT* Vehicle Miles Travelled, *US* United States, *TV* Television

#### Study design

Of the 19 included studies [18–36], nine were controlled interrupted time series (CITS) [18, 19, 23–26, 31, 32, 36], seven were uncontrolled interrupted time series (ITS) [20, 21, 27–30, 33] and three were controlled before-after studies (CBA) [22, 34, 35]. Nine [23–28, 31–33] of the studies had concomitant enforcement activities taking place at the time of the media campaign and the effect of the media campaign was not analyzed separately.

#### Participants

Seven studies [18, 19, 21, 22, 25, 28, 32] specified a target age group for their media campaigns, ranging from 15 to 34 years of age. One study [26] summarized a mixture of media campaigns that had both target age groups and no target age groups. The rest targeted drivers of all ages.

#### Settings

There was one study each from Thailand [33], Italy [34], and Australia [20]. Three were from New Zealand [21, 30, 36], and the rest were from the US.

#### Interventions

Media activities included advertisements in newspaper, radio, broadcast and cable television, cinema, billboards, posters, banners, stickers, with a combination of paid and earned media. There were no projects that explicitly described the use of social media in their campaigns. Those that had concomitant enforcement activities included interventions such as speed cameras, compulsory breath testing, sobriety checkpoints and patrols, changes in speed limits, driving under the influence (DUI) legislation or drinking age. Three studies [22, 23, 34] had a supplementary education program in the target community, including workplace [22] and school [34].

#### Comparator

Eleven studies [18, 19, 22–26, 31, 32, 35, 36] defined a comparator. One study used different hours in the day (“high alcohol consumption hours” and “low alcohol consumption hours”) for comparison [36]. The rest either compared different counties within the same state [18, 22–24, 35], neighboring states [19, 25, 31, 32], or data from the entire nation [26].

#### Outcome measures

**Fig. 3:**
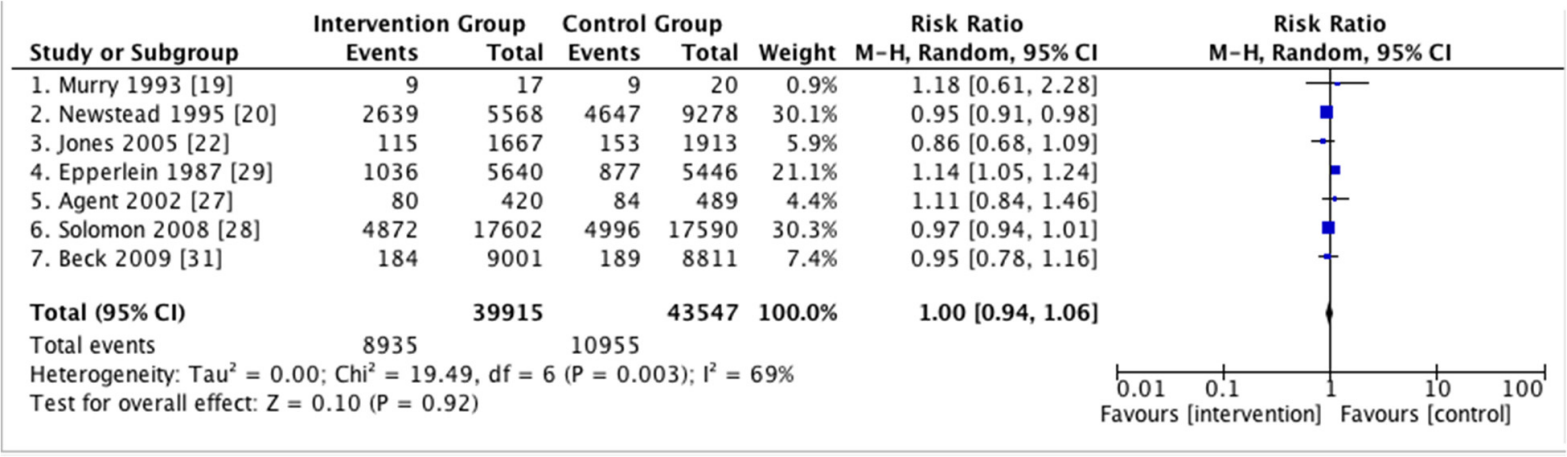
Pooled effects. Outcome measures used for summary effects calculation: 1. Murry 1993: Nighttime fatal and incapacitating accidents in 15 to 24-year-old males and females/ Total fatal and incapacitating accidents 15 to 24-year-old males and females. 2. Newstead 1995: Serious casualty crashes in all victoria during high alcohol hours /All hours. 3. Jones 2005: Nighttime single-vehicle crashes/ All crashes. 4. Epperlein 1987: Proportion of drinking drivers in crashes/ Total traffic crashes. 5. Agent 2002: Alcohol-related injuries or fatalities/Total number of crashes. 6. Solomon 2008: The number of motor vehicle fatalities for male drivers (BAC ≥0.08 g/dL) age 18 to 34/Total number of alcohol-related fatalities. 7. Beck 2009: Alcohol-related fatality crashes/ Alcohol-related total crashes

Eight studies [23–28, 31, 32] used alcohol-related fatal crashes as outcome measures. The rest used proxy measures for outcome (Fig. 3).

### Quality measures and risk of bias

See Tables 3 and 4. Based on the results of quality assessment, four studies were rated as good [18–21], five as intermediate [22–26], and eight as low [27–34]. The quality of two studies [35, 36] could not be assessed due to unavailability of the manuscript but they were included in this review as they were included in the review by Elder [8].

**Table 3:** Summary table on risk of bias of the included interrupted time series studies (excludes two studies (35, 36) that could not be assessed)

ITS	Intervention independent of other changes	Shape of the intervention effect pre-specified	Intervention unlikely to affect data collection	Knowledge of the allocated interventions adequately prevented during the study	Incomplete outcome data adequately addressed	Study free from selective outcome reporting	Study free from other risks of bias
Whittam 2006 [18]	Low risk (ARIMA model used and had comparator site)	Low risk	Low risk	Low risk	Low risk	High risk (crash data only for 16–19 year olds)	Low risk (has comparator site, using ARIMA model)
Good quality study							
Murry 1993 [19]	Low risk (authors state that data were transformed to isolate the experimental effect from any extraneous influences)	Low risk	Low risk	Low risk	Low risk	High risk (using proxy indicator, using certain age group only)	Low risk (using comparator site, using model)
Good quality study							
Newstead 1995 [20]	Low risk (regression model used to account for other factors)	Low risk	Low risk	Low risk	Low risk	Unclear risk (using proxy indicator)	Low risk (using regression model, but no comparator site)
Good quality study							
Tay 2002 [21]	Low risk (used regression models to exclude other factors)	Low risk	Low risk	Low risk	Unclear risk (States that some inconsistencies may exist in the reporting as done by local police)	Unclear risk (used proxy measures)	Low risk
Good quality study							
Fell 2008 [23]	High risk (other enforcement measures took place)	Low risk	Low risk	Low risk	Low risk	Low risk	Low risk
Intermediate quality study							
Zwicker 2007a [24]	High risk (enforcement also took place)	Low risk	Low risk	Low risk	Low risk	Low risk	Low risk (used ARIMA model and applied parameters to model periodic fluctuations in the crash rates)
Intermediate quality study							
Zwicker 2007b [25]	High risk (enforcement also took place)	Low risk	Low risk	Low risk	Low risk	Low risk	Low risk (contiguous county data were used to remove factors that may have obscured the effect of the campaign on the trend)
Intermediate quality study							
Lacey 2008 [26]	High risk (law enforcement activities also took place)	Low risk	Low risk	Low risk	Low risk	Low risk	Low risk (ARIMA model used)
Intermediate quality study							
Epperlein 1987 [29]	Unclear risk (no comparator site, but daytime crashes used to account for other changes)	Low risk	Low risk	Low risk	Low risk	Unclear risk (using proxy indicator)	High risk, not using model
Low quality							
Miller 2004 [30]	High risk (media campaign done together with other enforcements, though model was used to look at each interventions)	Low risk	Low risk	Low risk	Low risk	Unclear risk (using proxy indicator, fatal nighttime crashes)	High risk (ARIMA model was used, but evaluation of mixed approaches in different areas over different period)
Low quality study							
Agent 2002 [27]	High risk (enforcement activities also took place as part of the campaign)	Low risk	Low risk	Low risk	Low risk	Unclear risk (documentation of alcohol use is dependent on the reporting officer)	High risk (no model used)
Low quality study							
Solomon 2008 [28]	High risk (enforcement measures also in place)	Low risk	Low risk	Low risk	Low risk	Low risk	High risk (Only looking at changes in absolute numbers, no application of models, no comparator site)
Low quality study							
Beck 2009 [31]	High risk (enforcement also took place)	High risk (point of analysis not clear)	Low risk	Low risk	Low risk	Low risk	High risk (only looking at the absolute number of alcohol-related crashes, not using any models or accounting for rates in comparator sites)
Low quality study							
NHTSA 2007 [32]	High risk (enforcement also took place)	Low risk	Low risk	Low risk	Low risk	Low risk	High risk (compared with non-intervention sites, but no model used. Unclear if other factors accounted for)
Low quality study							
Suriyawongpaisal 2002 [33]	High risk (enforcement measures also used)	High risk (point of analysis is not the point of intervention, and not clearly stated why the data points were selected)	Low risk	High risk (hospital staff of the study sites were not blinded, and could have affected how they collected data)	High risk (not sure what proportion of cases were missed in each period, data collection dependent on hospitals enrolled)	Unclear risk	High risk (the study did not account for other changes that could have affected the outcome)
Low quality study							

*ARIMA*, Autoregressive Moving Average Model; *ITS*, Interrupted Time Series; *NHTSA*, National Highway Traffic Safety Administration

**Table 4:** Summary table on risk of bias of the included controlled before after studies (excludes two studies [35, 36] that could not be assessed)

CBA	Allocation sequence generation	Allocation adequately concealed	Baseline outcome measurements similar	Baseline characteristics similar	Incomplete outcome data adequately addressed	Knowledge of the allocated interventions adequately prevented	Study adequately protected against contamination	Study fee from selective outcome reporting	Study free from other risks of bias
Jones 2005 [22]	High risk	High risk	Low risk	Unclear	Unclear	Low risk	Unclear risk (selected Pueblo and surrounding counties as intervention sites, but possibility of contamination remains)	Unclear risk (using surrogate indicator)	Unclear risk (not sure if it has accounted for other changes during before/after)
Intermediate quality study									
Zampetti 2013 [34]	High risk	High risk	Low risk	Unclear	Unclear	Low risk	Unclear (due to nature of intervention)	Unclear (using proxy indicator)	High risk (has not taken into account other changes during study period)
Low quality study									

*CBA* Controlled Before-after

#### Studies with no increases in enforcement activities or with statistical models to account for those increases

Good quality: four studies [18–21] were included in this category. All except for one [21] had a comparator, and showed reduction in AID-related adverse outcome measures. Of those, the decrease reached statistical significance in two out of the three studies. While media campaign did not seem to show any impact in the target population (male 15–34 years old) in the study by Tay 2002 [21], it did show decreases in other age groups (male 35–54, females 15–34).

Intermediate quality: one study [22] was included in this category. Relative to the comparator counties, the intervention site had a statistically significant net decrease of nighttime single vehicle crashes by 28.8 % (−24.8 % in intervention group, +4.0 % in comparison group; *p* = 0.01) after the intervention. The study was classified as intermediate quality as the baseline characteristics in the intervention and comparator sites were not clearly addressed, and since there was a question about possible contamination of the effects and other biases due to the nature of the study (CBA studies).

Low quality: three studies were included in this category [29, 30, 34]. All three studies showed various degrees of decrease in the outcome measures, though only one [29] reached statistical significance. The studies were categorized into low quality primarily because there was no use of statistical models to assess the impact of the media campaigns.

In summary, studies that evaluated the impact of media campaigns with no increases in enforcement activities or with statistical models to account for those increases showed that the campaign resulted in a median decrease in the outcome measures by 15.1 % (range 0–28.8 %).

Quality not assessed: two studies were included in this category [35, 36]. The study by Worden et al. did not result in any net changes, but the sample size was small and the estimates were deemed to be unstable (*p* > 0.05). The study by Cameron et al. [36] comparing high alcohol hour to low alcohol hour showed a statistically significant decrease in serious injury crashes after intervention (net change −7 % in urban arm, −18 % in rural arm; *p* < 0.05 for both arms).

#### Studies with increases in enforcement activities but without statistical models to account for those increases

Good quality: there were no studies that were considered as good quality due to the classification criteria described in the methods section.

Intermediate quality: four studies were included in this category [23–26]. The study by Fell et al. [23] included results from seven states that used publicized enforcement along with various enforcement programs. From this study, it was concluded that the programs that experienced significant reductions included the use of paid media to publicize the enforcement, using a statewide model rather than selected portions of the state, and the use of highly visible and frequent sobriety checkpoints [23]. The remaining three assessed the results of projects that used a combination of media campaigns and sobriety checkpoints as enforcement measures. All three studies used alcohol-related fatal crashes as outcome measures and showed reduction, though the study by Lacey et al. [26] did not reach statistical significance.

Low quality: five studies were included in this category [27, 28, 31–33]. The intervention by Agent et al. [27] and Solomon et al. [28] were similar in that they both involved targeted media campaigns and enforcement measures surrounding the Labor day holiday. Both showed some degree of reduction in the outcome measures, although there were differences in the outcomes measured (alcohol-related fatalities during the year pre- and post- intervention versus alcohol-related crashes and injuries 13 days around Labor day). The study by Suriyawongpaisal [33] showed significant increase in the percent of drivers among traffic injury victims with illegal BAC (≥0.05 g/dL) among traffic injury cases (30.0 to 44.6 %, net change +14.6 %). However, it should also be noted that the methodology of this study was different from other studies in that: 1) the study was conducted after 8 months of law enforcement and 2 years of active public education program without baseline figures prior to the intervention, 2) the results did not take into account the changes in overall number of traffic accidents during the study period, and 3) data collection was done during a pre-defined period, therefore, prone to reporting bias.

In summary, studies that measured the effects of concomitant enforcement activities in addition to media campaigns showed a median reduction of 8.6 % (range −36.4 to +14.6 %) in their outcome measures.

### Summary effect measures

A total of seven studies [19, 20, 22, 27–29, 31] were included in the summary effect measures calculation, and the results are summarized in Fig. 1. Results of pooled analysis of the seven studies did not show any improved risk of alcohol-related injuries or fatalities from the intervention (RR = 1.00, 95 % CI = 0.94–1.06).

## Discussion

While results from individual studies suggested reduction in their respective outcome measures after intervention, reduction was not observed in the pooled analysis of relative risk of alcohol-related injuries or fatalities by media campaigns. This is likely due to the large heterogeneity observed in the methodology of the media campaigns, the follow-up methods, and the outcome measures used: Some studies had concomitant enforcement measures along with the media campaigns, and not all studies conducted analyses to examine the effects from media campaigns only; variety in the duration and intensity of media campaigns were observed; proxy measures were used in some studies for alcohol-related fatal crashes, and the presentation of outcome varied from mean cases per month generated from a model to raw figures based on changes in annual cases pre- and post- intervention. An attempt was made to include only those studies that allowed comparison of similar outcome measures (e.g. risk of alcohol-related fatalities over all crashes) in the summary effects calculation. Regardless, heterogeneity remained among the included studies.

### Messages used in media campaigns

Six out of the eight studies that assessed the effects of media campaigns independently showed statistically significant differences after intervention [19–22, 29, 30]. Some of these studies have attributed their success in their campaigns to having a message that emphasized the consequences of alcohol-induced driving. Examples include messages such as “Drunk Drivers Should Be Barred” [29] or “DUI: the $8866 Hangover” [22]. The later indicates the true cost of DUI as the sum of increasing insurance costs, lawyer fees, fines, and other expenses [22]. Miller et al. [30] did not describe the details of the media campaign, but they did state that the campaign was “harder hitting and more intensive” compared to previous campaigns. The purpose of the study by Tay et al. [21] was to examine the impact of a fear-based advertising campaign. While their analysis showed that the intervention was effective for certain age groups, it did not seem to influence the main target population, which was male 15–34 years old. Therefore, the authors concluded that, “an appeal to the emotion of fear will evoke different responses from different segments of an audience”.

This study built on the study by Elder 2004 [8], and added an additional decade of research literature. There are several limitations to this study: First, some eligible reports may have been missed due to language. Most of the studies included in this review are coming from English-speaking developed countries, namely, the US, Australia, and New Zealand. Therefore, the results may not be generalizable to low- and middle- income countries where traffic regulations and driving practices may be different. Second, most of the studies did not describe the interventions well enough for the reader to understand the intensity of mass media campaigns. This resulted in challenges and ambiguities in extracting data. Third, heterogeneity was large among the included studies, including settings, methods, and outcome measures used, as described earlier.

Although the pooled analysis did not show any evidence that media campaigns reduce the risk of alcohol-induced fatalities, we cannot conclude that media campaigns have no effect altogether given the large heterogeneity seen among studies. It is surprising that only a limited number of good quality study could be added to update the review of Elder et al. [8], considering the wider availability of options to conduct media campaigns. In addition, drink driving remains to be one of the leading causes of death in many countries and millions of dollars have been spent on mass media campaigns to reduce them.

## Conclusions

Heterogeneity in methodology, interventions and outcome measures were observed among the included studies and pooled analysis did not show evidence that media campaigns reduced the risk of alcohol-related fatalities.

More studies are required to find how mass media could be made more cost-effective in terms of timing and location, target audience, and message and campaign characteristics. In addition, more studies from low- and middle-income countries are needed where the majority of road traffic deaths occur.
